# Impact of the Temperature Reconditioning of Cold-Stored Potatoes on the Color of Potato Chips and French Fries

**DOI:** 10.3390/foods13050652

**Published:** 2024-02-21

**Authors:** Evelyne Nkirote Gikundi, Ariel Buzera, Irene Orina, Daniel Sila

**Affiliations:** 1 Graduate School of Animal Science and Agriculture, Obihiro University of Agriculture and Veterinary Medicine, Inadacho, Nishi 2 Sen-1, Obihiro 080-8555, Hokkaido, Japan; 2 School of Food and Nutrition Sciences, Jomo Kenyatta University of Agriculture and Technology, Nairobi P.O. Box 62000-00200, Kenya; arielbuzera@gmail.com (A.B.); irene.orina@jkuat.ac.ke (I.O.); dsila@jkuat.ac.ke (D.S.); 3 Faculty of Agriculture and Environmental Science, Universite Évangélique en Afrique, Bukavu P.O Box 3323, Sud-Kivu, Democratic Republic of the Congo

**Keywords:** potato, storage, sugars, reconditioning, cold storage

## Abstract

The effect of temperature reconditioning on cold-stored potato tubers was investigated for three popularly consumed potato varieties (Shangi, Unica, and Dutch robjin) grown in Kenya. The potatoes were stored at 4 °C for 30 days, followed by removal and storage at 22 ± 3 °C for 9 days during which changes in sugar concentration were evaluated every two days. In parallel, potato chips and French fries were processed, and their colors were determined. The results showed that sugar content decreased significantly with increasing reconditioning time. The relative decrease in fructose content was the highest (*p* < 0.05) in Dutch robjin (57.49%), followed by Shangi (49.22%) and Unica (38.18%). Glucose content decreased by 54.1% in Dutch robjin, 49.5% in Shangi, and 50.8% in Unica. The lightness (L*) of French fries and chips increased significantly (*p* < 0.05) with reconditioning time while the redness (a*) values decreased significantly (*p* < 0.05) across all varieties. The correlation between lightness and the total reducing sugar content of the potatoes was r < −0.93, indicating a strong negative correlation for both products. The coefficient of determination showed that the glucose content of the tubers accounted for 80.5–97.6% of the lightness of French fries and 88.4–94.2% for potato chips. The critical glucose content range for acceptable products in French fries and chips based on the color (L* and a*) values was 12–22 mg/100g and 8–14 mg/100g, respectively, for the varieties in this study.

## 1. Introduction

Potato plays a key role in the global sustainable food system, as it possesses a unique combination of characteristics such as a high yield, versatility in use, a low cost, and a good nutritional quality. The crop yields more food per unit area and time than any other food crop and can be processed into a wide array of food and non-food products [1]. Although potatoes are still largely consumed fresh in many countries, there is a rapid increase in demand for high-value-added convenient potato-based foods due to increasing urbanization [2,3]. Potato chips and French fries dominate the global snack food market [4,5]. In the United States, for instance, over 60% of potatoes are utilized in the processed form, with frozen fries accounting for more than half (41%) of the processed products [6]. The processing and consumption of French fries in Kenya has accelerated as evidenced by the spread of eateries serving fries, chicken, and soft drinks [7]. Germany, Italy, Belgium, France, and Spain are some of the leading countries in the consumption of potato chips [8]. The quality of the processed products in terms of appearance, flavor, and texture, among others, is largely influenced by post-harvest management practices and the related intrinsic properties of potato tubers [9,10]. Additionally, pre-harvest practices and factors such as cultivar, genetic improvement, fertilization, growing season (e.g., spring, autumn), and tuber maturity play a significant role in the quality of processed potato products [11,12,13,14,15,16,17]. 

The successful long-term storage of fresh potatoes is often challenging due to the biochemical and physical changes that occur in the tuber, including sprouting, shrinking, greening, and rotting, among others. Typical post-harvest potato storage practices include temperature (0–10 °C) and relative humidity (≥90%) control, use of sprout suppressants, ethylene treatment, and storage in well-ventilated dark places, among other practices [18,19,20,21]. The use of chemical suppressants has recently aroused concerns over environmental safety and toxicity, and their use has been banned in many countries following concerns of chronic consumer risk [22,23,24]. Reliance on non-chemical options, mainly storage at colder temperatures even below 4 °C, has, thus, been increasing gradually [25]. Although low-temperature storage (4–10 °C) has proven effective in preserving the quality of potato tubers in terms of inhibiting sprouting, weight loss, rotting, and greening, it also leads to the accumulation of reducing sugars such as fructose, glucose, and sucrose in a process called cold-induced sweetening (CIS) [26]. 

Cold-induced sweetening causes significant losses to the potato processing industry because these sugars react with free amino acids during frying in a process called the Maillard reaction, leading to the production of dark-pigmented products with an off-flavor, bitter taste, and unsightly appearance to consumers [25]. Moreover, the Maillard reaction leads to the formation of acrylamide, a compound classified as a neurotoxin and a probable carcinogen [27]. CIS is, thus, a critical parameter considered during potato storage and, therefore, measures to mitigate its effects are deemed necessary [28]. Potato tuber reconditioning, a process entailing exposing potatoes to higher temperatures (>20 °C) post cold storage for a period of time [29], has gained attention as a potential approach to mitigate the effects of CIS. This process aims at restoring the sugar balance in potatoes after cold storage, thereby enhancing product quality, especially color [30].

**Figure 1:**
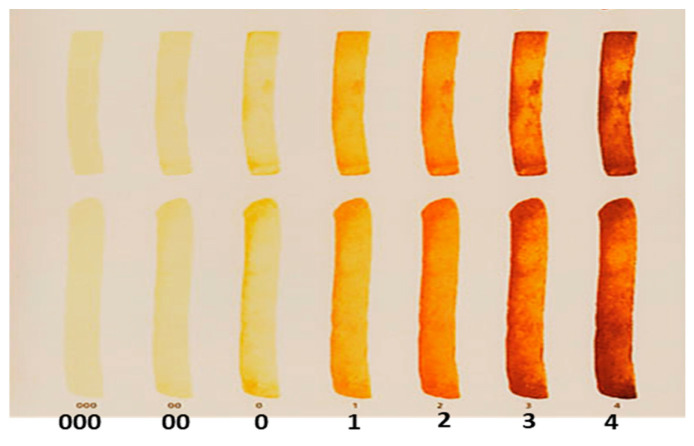
USDA Munsell color chart for evaluating commercial frozen French fry color (USDA, 1967).

The color of a food’s surface is paramount because it is the first parameter evaluated by consumers, therefore, determining its acceptance even before it enters the mouth [31]. Regardless of the method of preparation of ready potato snacks, they should be light and golden, without brown discoloration, black streaks, or stains [16,17,32]. According to the European Potato Processors Association, the acceptable color for fried potato products is a light golden color (corresponding to No. 0 on the USDA Munsell color chart) or golden yellow (corresponding to No. 1 on the USDA Munsell color chart), as shown in Figure 1 [33]. Although this chart (Figure 1) was designed to evaluate the color of French fries, it has also been used successfully to assess the color of potato chips [34]. This approach is empirical and largely subjective depending on the user.

This study aimed to understand the impact of potato tuber reconditioning on the levels of reducing sugars and sucrose and the related color changes on fried potato products (potato chips and French fries), with an overall goal of developing a simple quantitative approach towards grading potato-based products.

## 2. Materials and Methods

### 2.1. Sample Acquisition and Preparation

**Figure 2:**
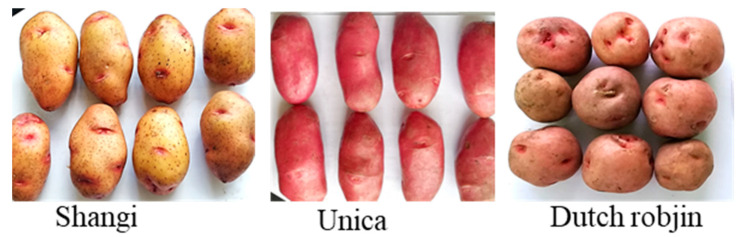
Shangi, Unica, and Dutch robjin potato varieties used in this study.

Shangi, Unica, and Dutch robjin potato varieties were cultivated under similar agronomic conditions and cultural practices on a farm (Jaconta FarmsFarms Limited) in Nyandarua County, Kenya (0°14′55″ S 36°26′27″ E). Cultivation was performed during the short rainy season (October 2021–January 2022) following standard practices comprising ridging, fertilization (Diammonium phosphate during planting), weeding, and pest control. Shangi (oval shape, yellow skin, and flesh color) is the most popular and cultivated variety in Kenya, mainly used for French fry processing and other homemade dishes. Unica is a fairly new variety with a red skin and yellow flesh used for chip and French fry processing as well as table use, while Dutch robjin is rounder in shape with a red skin and white flesh and is popular for potato chip processing [10,35]. Figure 2 illustrates the three potato varieties. The potatoes were manually harvested and immediately transported in nylon sacks at room temperature to Jomo Kenyatta University of Agriculture and Technology, Kenya. The tubers were left in a dark room to cure for 1 week at room temperature after which sorting was performed, and medium-sized (length 55–95 mm), undamaged, and apparently healthy tubers were selected for the experiment. The potatoes were then stored in a cold room at 4 °C for 30 days. The tubers were then removed out of the cold store and kept at room temperature (22 ± 3 °C) for 9 days. During this period, a random sampling of the potatoes was performed every 2 days, and the samples were used for the analysis of reducing sugars and sucrose as well as preparation of potato chips and French fries. In all the potato products, the color was determined and paralleled by taking photographs. 

### 2.2. Determination of Glucose, Fructose, and Sucrose Contents

The concentration of sugars (glucose, fructose, and sucrose) was determined using the method described by Gikundi et al. [26], whereby 5 g of potato tubers freshly homogenized using a blender was weighed into 50 mL pear-shaped flasks. Twenty (20) ml of ethanol was added to the flasks and swirled to mix. The mixture was refluxed at 100 °C for 1 h after which the contents were filtered to obtain the filtrate. The filtrate was evaporated at 80 °C to dryness on a rotary evaporator, after which a solution of acetonitrile and distilled water (2 mL) in the ratio of 1:1 was added to reconstitute the dried sample. The mixture was passed through 0.45 μm pore size micro-filters to eliminate any debris before injecting 20 µL into a Shimadzu Nexera Ultra-Flow Liquid Chromatography machine (UFLC, Kyoto Japan) fitted with a SIL-20A HT prominence autosampler, refractive index detector-20A, and an LC-20AD pump. The chromatographic conditions entailed an isocratic elution of water and acetonitrile (25:75) pumped through a normal phase Ultisil NH_2_ column with a 6 × 250 mm internal diameter at a flow rate of 1.8 mL/min. The column oven (CTO-10ASvp) temperature was set at 40 °C. Glucose, fructose, and sucrose were quantified by comparing them against their corresponding standard solutions and expressed as mg/100 g fresh weight.

### 2.3. Preparation of Potato Chips and French Fries

Potato chips and French fries were prepared following the process described by Abong’ and Kabira [36]. The potatoes were peeled and then uniformly sliced into approximately 1.5 mm slices for potato chip processing or cut into French fry sticks (10 mm thickness) using a manual hand-operated chipper and French fry cutter, respectively. The slices were washed in tap water to remove surface starch and, thereafter, gently shaken on a mesh sieve to drain excess water. Using a portable electric deep fryer (Ramtons stainless steel-RM/370 with a temperature control function), the chip slices were fried at 180 °C in corn oil for 3 min and removed. After frying, excess oil was drained off for about 2 min, and the chips were left to cool. French fries were processed by frying at the temperature of 180 °C for 8 min, and the excess oil was drained off. After cooling, the color of the potato chips and French fries was analyzed. 

### 2.4. Determination of Color

The color of the potato chips and French fries was determined using HunterLab’s ColorFLex EZ spectrophotometer using the CIE Lab L*a* and b* color scales as described by Buzera et al. [37]. The instrument was first calibrated using white and black tiles as the standards. The chips and French fries were tightly packed in an optically transparent glass cup and covered with another opaque cover used as a light trap to prevent the external light from interfering with the sample cup. The cup was placed on the port, and the color intensity was measured on the following rating scale: lightness L* ranging from black (0) to white (100), with a* representing the chromatic red–green parameter; where positive (a*+) and negative (a*-) indicate red and green values, respectively, and b* represents the yellow and blue color components as positive (b*+) and negative (b*-), respectively. The color difference ∆E between the initial and final products after the reconditioning period was determined using Equation (1), where ∆L*, ∆a*, and ∆b* are the differences in the initial and final attribute values.

(1) $$∆E=\sqrt{∆L^{*2}+{∆a}^{*2}+{∆b}^{*2}}$$

### 2.5. Image Acquisition

After frying and cooling, the potato chips and French fries were photographed against a white background for a visual confirmation of the color changes with reconditioning time.

### 2.6. Statistical Analyses

The data are reported as means of triplicate observations and are expressed as mean ± standard deviation. Analysis of Variance (ANOVA) and Bonferroni’s test at the significance level of 0.05 were conducted to evaluate the significant differences among sample means using STATA software version 17.0 (StataCorp LLC, College Station, Texas Tx, USA). Correlation analysis was performed using Microsoft Excel (Ms Office 2021). All the measurements were performed in triplicates.

## 3. Results and Discussion 

### 3.1. Changes in Fructose, Glucose, and Sucrose Concentration with Reconditioning Time

**Figure 3:**
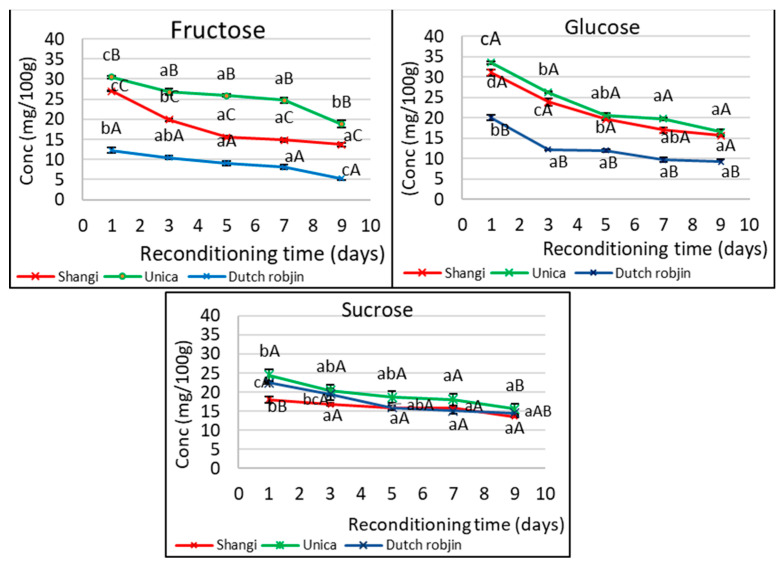
Changes in the concentration of glucose, fructose, and sucrose in cold-stored potato tubers after reconditioning at room temperature (22 ± 3 °C for 9 days). Different lower-case letters indicate significant differences across the days in reconditioning while upper-case letters indicate significant differences across the varieties based on Bonferroni’s means separation test at a 5% level of significance.

The content of sugars (fructose, glucose, and sucrose) reduced significantly with reconditioning time in all the potato varieties (Figure 3). Additionally, the concentration of the sugars was significantly different among the three varieties (*p* < 0.05%). A significant reduction in the level of fructose (*p* < 0.0001) was observed in all the varieties. The relative decrease in fructose content was highest (*p* = 0.0011) in Dutch robjin (57.49%), followed by Shangi (49.22%), and then Unica (38.18%). A similar trend was observed for glucose, whereby a significant reduction with reconditioning time was observed across all the varieties: (*p* = 0.0002) in Dutch robjin, (*p* < 0.0001) in Shangi, and (*p* < 0.0001) in Unica. Dutch robjin exhibited a 54.14% reduction in glucose levels while Shangi and Unica exhibited a 49.49% and 50.77% reduction, respectively. However, there was no significant difference (*p* = 0.2495) in the relative decrease in glucose concentration across the varieties. The tubers also showed a significant decrease in sucrose content with reconditioning time in all the varieties: (*p* = 0.0002) in Dutch robjin, (*p* = 0.0010) in Shangi, and (*p* = 0.0036) in Unica. The relative decrease in sucrose content was significantly different among the varieties, with the highest rate being observed in Unica (36.51%) followed by Dutch robjin (35.35%) and Shangi (24.86%). 

These findings are in agreement with the trend reported by Zommick et al. [38], whereby tubers of American potato cultivars stored at 4 °C for 30 days and then reconditioned at 16 °C for 3 weeks exhibited a significant decline in reducing sugar levels. Similarly, according to a study by Kumar and Ezekiel [39], potatoes stored at 10–12 °C exhibited a decline in reducing sugars upon reconditioning at 20 ± 2 °C for 1–2 weeks. The cultivar with the highest accumulated reducing sugar level (333 mg/100 g) showed a reduction to 32 mg/100 g after 2 weeks of reconditioning [39]. Wayumba et al., (2019), reported that after storage at 4 °C, reconditioning 27 South Korean potato clones at an ambient temperature of 22 °C for 21 days led to a reduction in total reducing sugars, whereby the lowest reduction was by 2.2% while the highest reduction was by 86.1%.

Among other factors, the level of reducing sugars is considered one of the most limiting parameters in the processing of fried potato products after cold storage. Research has shown that storing potatoes at low temperatures leads to the accumulation of reducing sugars with detrimental effects on the quality of fried potato products [40]. The accumulation of reducing sugars occurs due to starch breakdown occurring hydrolytically or phosphorytically into sucrose, glucose, and fructose [41]. It is suggested that hexose phosphates (products of starch breakdown from the amyloplasts) are restricted from entering the glycolytic pathway due to the inactivation of glycolytic enzymes such as fructose-6-phosphatase and phosphofructokinase by the low storage temperature [41,42]. The hexose phosphates and other starch metabolites are, thus, rerouted to the sucrose synthesis pathway. Here, they are converted into sucrose by the enzyme sucrose phosphate synthase, after which sucrose may be subsequently hydrolyzed to glucose and fructose by the vacuolar acid invertase (VINV) enzyme [12,43].

Reconditioning is believed to reduce the level of reducing sugars through the respiration and reconversion of the free sugars into starch [29]. The biochemical process involved in reconditioning has not been investigated in detail. However, it is suggested that at higher temperatures, the enzymes responsible for sugar accumulation in cold storage are suppressed, while the enzymes involved in the conversion of sucrose to starch may be stimulated. Approximately 80% of the reducing sugars are reconverted to starch, and the remaining 20% are degraded through respiration [44]. As demonstrated in this study, the effectiveness of reconditioning in restoring tuber quality in terms of reducing sugars is variety-dependent. The difference in the rates of decrease in the sugar content could be attributed to genotypic variations in starch degradation metabolism, sucrose content in tubers at harvest, the activity of major enzymes such as invertase, and the expression of vacuolar invertase inhibitor which regulates the activity of invertase enzyme [30,45]. 

### 3.2. Evolution of Color in Potato Chips and French Fries

The lightness (L*) of the potato chips and French fries increased significantly with increasing reconditioning time in all the varieties, as shown in Table 1. In the potato chips, the lightness score ranged between 43.26 and 49.85 across the varieties for potato chips processed on the first day post cold storage. By the 9th day of reconditioning, the lightness scores ranged from 64.93 to 68.12 in all the varieties, an increase of 55.8% in Shangi, 50.1% in Unica, and 34.0% in Dutch robjin. The relative increase in lightness was significantly lower in Dutch robjin compared to Shangi and Unica (*p* = 0.0065). The lightness (L*) index is a critical parameter in the frying industry as it is generally the first attribute perceived by consumers when gauging product quality. The L* parameter shows the degree of the whiteness of products and ranges from 0 (black) to 100 (white). Lower L*values indicate that a dark color is mainly associated with non-enzymatic browning reactions, contributing to undesirable French fry and potato chip colors [6,46].

The redness values (a*) of the chips decreased significantly in all the varieties. On the first day of potato chip processing post cold storage, the redness values ranged from 10.98 to 14.54. Considering all the varieties, these values (a*) dropped to 3.29-5.67 by the last day of tuber reconditioning with the lowest decrease observed in Unica (48.34%) (*p* = 0.0117) while Dutch robjin (77.39%) and Shangi (73. 09%) did not differ significantly. The redness component (a*) is an indicator of non-enzymatic browning and has been shown to be positively correlated to acrylamide content (a probable carcinogen resulting from the Maillard reaction during the frying process) [6]. The lower the a* value, the paler the potato chip color [47,48]. The yellowness (b*) attribute increased for Shangi (23.83%) and Dutch robjin (10.47%) potato chips, whereas Unica did not display any significant differences in b* values with reconditioning. 

As indicated earlier, similar to the potato chips, the lightness (L*) of French fries significantly increased with reconditioning time in all the varieties. The L* values of French fries processed after the first day ranged from 52.36 to 59.90. By the last day of reconditioning, the lightness values of the varieties ranged between 67.16 and 74.40, an increase of 28.77%, 28.27%, and 24.21% in Shangi, Unica, and Dutch robjin, respectively. The increase was significantly different among the varieties (*p* = 0.0064). On the other hand, the redness values of the French fries ranged from 6.67 to 8.85 among the varieties on day 1, before reducing significantly by 81.13% (Shangi), 77.59% (Unica), and 73.01% (Dutch robin) by the ninth day. This indicates a marked decrease in the darkness of the fries, which might lead to an increased acceptability of the fries in terms of color. The changes in the a* values did not differ significantly among the varieties (*p* = 0.1203). There was no significant difference in the yellowness (b*) values throughout the reconditioning period for all the varieties. The total color difference (∆E) in potato chips before and after reconditioning was 27.51 (Shangi), 24.40 (Unica), and 20.61 (Dutch robjin) while that in French fries was 19.07 (Shangi), 16.47 (Unica), and 16.65 (Dutch robjin). The ∆E of potato chips and French fries was not statistically significant among the varieties (*p* = 0.1590) and (*p* = 0.6927), respectively. The total color difference measured on a scale of 0 (less color difference) to 100 (complete color distortion) is an outcome of changes in the L*a*b* attributes, thus indicating the magnitude of color change [49]. Values ≤ 1.0 are not perceptible by the human eye, ∆E values > 2 mean that the difference is perceptible at a glance, and a value of 100 means that the colors being compared are exactly opposite [50,51]. It is an important tool for evaluating the perceptibility and acceptability of colors and for ensuring consistency in the colors of processed products [50]. 

**Figure 4:**
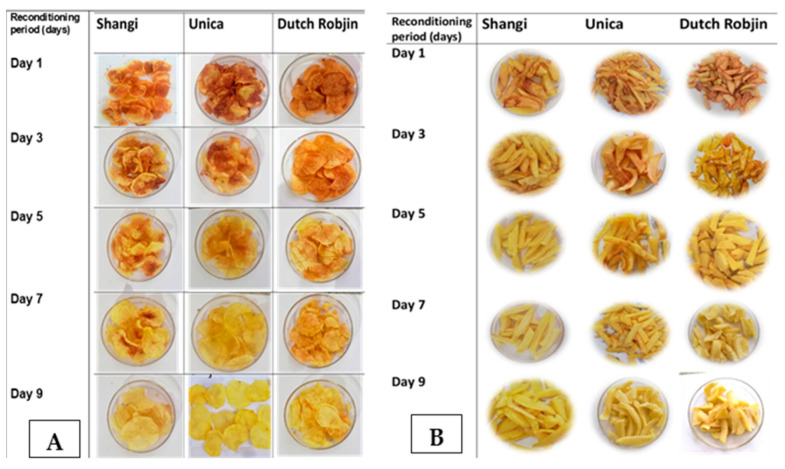
Evolution of the color of potato chips (**A**) and French fries (**B**) processed using cold-stored- then-reconditioned potatoes over time.

The consumer acceptability of potato chips and French fries is largely dependent on the color after frying [52]. Moreover, the color control of final fried potato products is recommended not only for consumer acceptability (appearance and sensory quality) but also to reduce acrylamide formation [6]. The color of fried potato products serves as an indicator of the extent of the Maillard reaction, which contributes to the formation of acrylamide. Lighter-colored products are generally perceived as more acceptable and safer due to lower acrylamide levels, whereas darker-colored products signify a higher degree of acrylamide formation and may raise concerns regarding safety [16,53]. Figure 4 shows the evolution of the color of potato chips and French fries as affected by reconditioning for 9 days. It is evident that the potato chips and potato fries became generally desirable after 5 days of reconditioning, and this was variety-dependent.

A study by Wayumba et al. [9] revealed similar trends to this study, whereby reconditioning cold-stored tubers had a positive effect on the potato chip color of most of the potato cultivars. The color of French fries from two American potato cultivars stored at 4 °C and then reconditioned at 16 °C improved by 10–19%, followed by a decline in reducing sugar concentration to basal levels (concentration before cold-induced sweetening) [38]. Similar findings were reported by Jaiswal et al. [54], whereby considerable improvement in the potato chip color of genetically modified Indian potato cultivars was observed upon room-temperature reconditioning (21 days) after storage at 4 °C for 30 days. Abong et al. [55], however, reported that tuber reconditioning for 3 weeks at 15 °C after storage at 4 °C for 3 months did not effectively improve the color of chips from eight Kenyan potato cultivars. This difference in observations could probably be attributed to the differences in variety and the storage conditions used. In addition to varietal differences and geographical variations during cultivation, the effectiveness of tuber reconditioning is significantly influenced by not only the period and temperature of reconditioning but also the cold-storage duration [30,38]. 

As indicated earlier (Figure 1), and according to the European Potato Processors Association, the acceptable color for fried potato products is a light golden color. In the present study, based on visual analyses, French fries and chips processed on days 5 and 9, respectively, were generally light golden in color irrespective of the variety. The corresponding ranges of the lightness and redness values for the three varieties were 64.67–68.19 and 2.82–3.10, respectively, for French fries, whereas the lightness and redness values for potato chips were in the range of 64.93–68.12 and 3.29–5.67, respectively. Although a similar trend in color evolution was observed for both the potato chips and French fries, the desired color was achieved sooner in French fries than in potato chips. This could be attributed to the difference in thicknesses [56,57] of the two products despite the less frying time for potato chips due to slice thickness. This suggests the potential need to lower either the frying temperature or duration, in addition to reducing sugars, to attain the acceptable potato chip color. 

### 3.3. Correlation between Reducing Sugars and the L* and a* Values of Fried Potato Chips and Potato Fries

**Figure 5:**
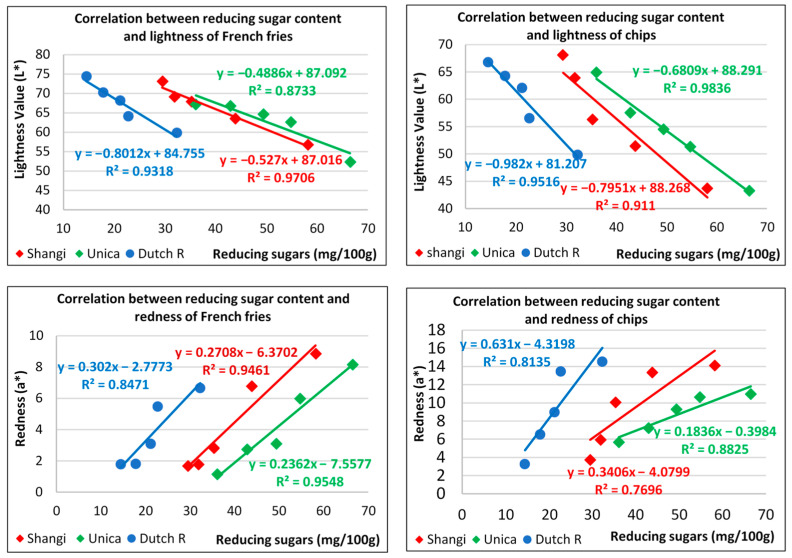
Correlation between reducing sugars and the lightness (L*) and redness (a*) of fried potato products.

The coloration of fried potato products is said to be influenced by several variables including growing conditions, tuber moisture content, and the frying time and temperature, but the sugar content stands out [9,58]. With increasing reducing sugar content in potatoes, the color of fried products deteriorates towards brown, and the acrylamide content also increases [53]. In this study, a strong negative correlation (r < −0.93) was observed between the reducing sugar concentration and lightness values of French fries and potato chips within the reconditioning time for all the potato varieties (Figure 5). The coefficient of determination indicated that reducing sugar concentration accounted for 87.3–97.1% of the variation in the L* parameter of French fries and 91.1–98.4% in potato chips. On the other hand, reducing sugar content was found to be positively correlated with the redness (a*) value of French fries (r > 0.92) and potato chips (r > 0.88). The coefficient of determination between reducing sugar content and the a* values ranged from 84.7 to 95.5% in French fries and 77 to 88.3% in chips. Jin et al. and Zommick et al. [38,59] reported that reconditioning cold-stored potatoes led to a reduction in their reducing sugar content and substantially improved the L* and a* values of potato-based products. These parameters are important because they are not only linked to flavor and appearance but also to the safety of fried potato products [16]. These results point to the fact that the reducing sugar content of cold-stored potatoes is a critical control factor for determining the quality of potato-based products. 

**Figure 6:**
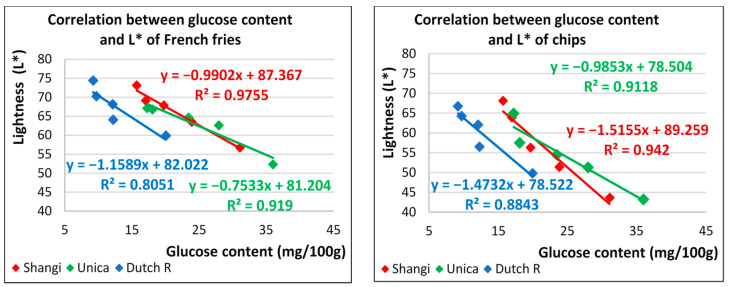
Correlation between glucose content and the lightness (L*) of French fries and potato chips.

To deepen the understanding, the correlation between the specific reducing sugars and the color of potato products was further explored. A strong negative correlation was observed between the glucose concentration and lightness values of French fries (r = −0.99 (Shangi), −0.96 (Unica), −0.90 (Dutch robjin)) and potato chips (r = −0.97 (Shangi), −0.95 (Unica), −0.94 (Dutch robin)) from all the potato varieties (Figure 6). The coefficient of determination indicated that glucose concentration accounted for 80.5–97.6% of the variation in the L* parameter of French fries and 88.4–94.2% in potato chips. This indicates that a simple quantitative method for predicting the color of potato-based products can be obtained based on a specific simple sugar concentration, for this case, glucose.

In the current study, a 5-day reconditioning period was considered to be equivalent to the minimum acceptable color threshold for potato fries corresponding to Figure 1. The maximum glucose level corresponding to the acceptable color in terms of L* and a* values for French fries was less than or equal to 22 mg/100 g per fresh sample irrespective of the variety. However, it should be noted that it is possible to develop variety-specific linear models depending on the sugar concentration of the potatoes and the reconditioning temperature. In potato chips, the same approach and model can be used, but better potato chip quality is obtained on day 9 with the highest glucose threshold being observed in Shangi (14 mg/100 g, worst-case scenario), thus making this the critical glucose level for high-quality chips production. Overall, this means that the glucose concentration could be used as an index for potato fries and chips quality.

## 4. Conclusions

Despite leading to the accumulation of sugars, the cold storage of potatoes is almost indispensable. Cost-effective methods are, thus, imperative to restore the processing quality of tubers. This study evaluated the effectiveness of the temperature reconditioning of cold-stored potato tubers at room temperature on the sugar content of the potatoes and the subsequent quality of processed French fries and potato chips in terms of color. The reconditioning process at room temperature (22 ± 3 °C) for up to nine days yielded significant reductions in the sugar content of the cold-stored tubers. This was accompanied by notable improvements in the color of French fries and potato chips as evidenced by the increase in lightness values and decrease in the redness values with reconditioning time. Generally, the yellowness attribute of the potato chips and fries did not change with reconditioning. Five days of reconditioning were sufficient to achieve an acceptable light color for French fries while potato chips needed nine days to achieve acceptable lightness for the varieties under this study. This suggests that the temperature reconditioning of potatoes after cold storage can restore the processing suitability of potatoes by reducing the sugar concentration.

In terms of variety, differences were observed in the rate of fructose and sucrose reduction, with Dutch robjin displaying the highest decrease in fructose content while Unica had the highest reduction of sucrose. Dutch robjin exhibited the lowest change in terms of the lightness of both products while Unica and Shangi showed no differences in the relative change in lightness. The relative change in redness was lowest in Unica’s potato chips while French fries showed no significant differences in this aspect across varieties. Despite these variations, the overall color difference ∆E was consistent among the three varieties. A correlation analysis showed that the reducing sugar concentration of the raw tubers strongly accounted for changes in the lightness and redness aspects of the French fries and potato chips. This study underscores the potential of reducing sugar content as a quantitative predictor and grader of potato-based products irrespective of the variety. Moreover, glucose concentration provides a simpler yet effective model to adequately predict potato-based product quality. 

## Figures and Tables

**Table 1:** Changes in the L*, a*, b* values in potato chips and French fries with post-cold-storage tuber reconditioning.

Time (Days)	1	3	5	7	9	*p*-Value
Parameter						
Potato Chips	Shangi					
L*	43.72 ± 1.95 a	51.46 ± 1.20 b	56.32 ± 3.13 b	63.91 ± 2.02 c	68.12 ± 1.95 c	<0.0001
a*	14.11 ± 1.62 ac	13.35 ± 0.84 c	10.05 ± 2.26 b	5.95 ± 1.15 ab	3.74 ± 0.31 ca	<0.0001
b*	29.38 ± 0.17 a	34.63 ± 1.11 b	36.13 ± 0.32 bc	38.59 ± 2.11 c	36.38 ± 1.49 bc	0.0001
∆E					27.51 ± 3.72 A	
Unica						
L*	43.26 ± 3.21 a	51.34 ± 2.52 b	54.54 ± 1.55 bc	57.55 ± 2.74 c	64.93 ± 1.47 d	<0.0001
a*	10.98 ± 1.35 b	10.64 ± 2.09 b	9.29 ± 3.22 ab	7.23 ± 0.54 a	5.67 ± 1.20 a	0.0129
b*	28.91 ± 4.69 a	32.99 ± 4.42 a	34.89 ± 0.87 a	36.92 ± 1.97 a	36.72 ± 3.29 a	0.0775
∆E					24.40 ± 4.74 A	
Dutch robjin						
L*	49.85 ± 2.11 a	56.54 ± 1.82 b	62.10 ± 3.68 bc	64.29 ± 1.48 c	66.78 ± 2.53 c	<0.0001
a*	14.54 ± 0.51 d	13.46 ± 1.28 cd	8.99 ± 2.95 bc	6.56 ± 3.06 ab	3.29 ± 0.64 a	0.0001
b*	34.85 ± 2.12 a	37.41 ± 0.27 ab	38.47 ± 2.94 ab	42.73 ± 0.99 b	38.50 ± 3.60 ab	0.0145
∆E					20.61 ± 2.05 A	
French fries	Shangi					
L*	56.76 ± 070 a	63.53 ± 3.55 ab	67.91 ± 2.11 bc	69.14 ± 1.30 bc	73.09 ± 3.56 c	0.0001
a*	8.85 ± 3.33 c	6.78 ± 2.28 bc	2.82 ± 0.33 ab	1.78 ± 0.43 a	1.67 ± 0.34 a	0.0006
b*	35.72 ± 3.18 a	36.29 ± 4.23 a	32.26 ± 2.90 a	37.94 ± 1.87 a	37.14 ± 2.76 a	0.7006
∆E					19.07 ± 2.33 A	
Unica						
L*	52.36 ± 2.35 a	62.60 ± 3.65 b	64.67 ± 2.50 b	66.80 ± 0.09 b	67.16 ± 3.11 b	<0.0001
a*	8.39 ± 0.82 c	5.98 ± 2.95 bc	3.10 ± 2.19 ab	2.73 ± 1.52 ab	1.88 ± 0.53 a	0.0007
b*	32.50 ± 3.32 a	38.00 ± 2.06 a	33.74 ± 3.90 a	32.33 ± 3.01 a	33.1 ± 3.56 a	0.0851
∆E					16.47 ± 4.81 A	
Dutch robjin						
L*	59.90 ± 4.10 a	64.13 ± 3.01 ab	68.19 ± 3.70 abc	70.28 ± 1.90 bc	74.40 ± 4.18 c	0.0005
a*	6.67 ± 2.89 b	5.48 ± 2.17 ab	3.10 ± 2.05 ab	1.82 ± 0.52 a	1.80 ± 0.33 a	0.0032
b*	37.70 ± 2.77 a	39.87 ± 3.66 a	35.96 ± 2.31 a	37.46 ± 1.09 a	34.58 ± 3.08 a	0.3785
∆E					16.65 ± 4.60 A	

Values are means ± standard deviations. Mean values with different lowercase letters on the same row are significantly different and mean values with different uppercase letters on the same column are significantly different based on Bonferroni’s means separation test at a 5% level of significance. *n* = 3.

## Data Availability

The data used to support the findings of this study can be made available by the corresponding author upon request.

## References

[1] Asnake D., Alemayehu M., Asredie S. (2023). Growth and tuber yield responses of potato (*Solanum tuberosum* L.) varieties to seed tuber size in northwest highlands of Ethiopia. Heliyon.

[2] Hussain T. (2016). Potatoes: Ensuring food for the future. Adv. Plants Agric. Res..

[3] Adekanmbi T., Wang X., Basheer S., Nawaz R.A., Pang T., Hu Y., Liu S. (2023). Assessing Future Climate Change Impacts on Potato Yields—A Case Study for Prince Edward Island, Canada. Foods.

[4] Riaz M.N. (2016). Snack Foods: Processing. Encyclopedia of Food Grains.

[5] Lal M.K., Tiwari R.K., Jaiswal A., Kumar R. (2023). Post-harvest management and value addition in potato: Emerging technologies in preserving quality and sustainability in potato processing. Indian J. Agron..

[6] Mesias M., Delgado-Andrade C., Holgado F., González-Mulero L., Morales F.J. (2021). Effect of consumer’s decisions on acrylamide exposure during the preparation of French fries. Part 2: Color analysis. Food Chem. Toxicol..

[7] George A., Wandayi O.M., Miriti J.W., Kabira J.N. (2021). Acrylamide intake in Nairobi Kenya: A case of French fries consumers. East Afr. J. Sci. Technol. Innov..

[8] Index Box (2021). The European Potato Chips Market Retains Growth Despite the Pandemic. Global Trade Magazine. https://www.globaltrademag.com/the-european-potato-chips-market-retains-growth-despite-the-pandemic/.

[9] Wayumba B.O., Choi H.S., Seok L.Y. (2019). Selection and evaluation of 21 potato (*Solanum tuberosum*) breeding clones for cold chip processing. Foods.

[10] Gikundi E.N., Sila D.N., Orina I.N., Buzera A.K. (2021). Physico-chemical properties of selected Irish potato varieties grown in Kenya. Afr. J. Food Sci..

[11] Byrne S., Meade F., Mesiti F., Griffin D., Kennedy C., Milbourne D. (2020). Genome-Wide Association and Genomic Prediction for Fry Color in Potato. Agronomy.

[12] Wu L., Bhaskar P.B., Busse J.S., Zhang R., Bethke P.C., Jiang J. (2011). Developing cold-chipping potato varieties by silencing the vacuolar invertase gene. Crop Sci..

[13] Khalil T., Haroon M., Miskeen S., Sammi S., Jahangir M., Najeeb S., Nisar K., Khan A., Liaquat M., Khan I. (2023). Potato Chip Varietal Analysis: A Comparative Evaluation Based on Potato Cultivars. Potato Res..

[14] Sharkar M., Ahmed J.U., Ahmed S.F., Al- Meraj S.M.Z., Mohi-Ud-Din M. (2019). Effect of harvesting dates on the yield and tuber quality of processing potatoes. Bangladesh J. Agric. Res..

[15] Freitas S.T., Pereira E.I.P., Gomez A.C.S., Brackmann A., Nicoloso F., Bisognin D.A. (2012). Processing quality of potato tubers produced during autumn and spring and stored at different temperatures. Hortic. Bras..

[16] Tajner-Czopek A., Kita A., Rytel E. (2021). Characteristics of french fries and potato chips in aspect of acrylamide content—Methods of reducing the toxic compound content in ready potato snacks. Appl. Sci..

[17] Nairfana I., Nikmatullah A., Sarjan M., Kisman (2021). Tuber and Organoleptic Characteristics of Four Potato Varieties Grown Off-season in Sajang Village, Sembalun. IOP Conf. Ser. Earth Environ. Sci..

[18] Murigi W.W., Nyankanga R.O., Shibairo S.I. (2021). Effect of Storage Temperature and Postharvest Tuber Treatment with Chemical and Biorational Inhibitors on Suppression of Sprouts During Potato Storage. J. Hortic. Res..

[19] Thoma J., Zheljazkov V.D. (2022). Sprout Suppressants in Potato Storage: Conventional Options and Promising Essential Oils—A Review. Sustainability.

[20] Wasukira A., Walimbwa K., Wobibi S., Owere L., Naziri D., Parker M. (2017). Ware Potato Harvesting and Storage Techniques: Guidelines for Harvesting and Storage Management of Ware Potato.

[21] Daniels-lake B.J. (2013). Carbon Dioxide and Ethylene Gas in the Potato Storage Atmosphere and their Combined Effect on Processing Colour. Ph.D. Thesis.

[22] Arena M., Auteri D., Barmaz S., Bellisai G., Brancato A., Brocca D., Bura L., Byers H., Chiusolo A., Marques D.C. (2017). Peer review of the pesticide risk assessment of the active substance chlorpropham. EFSA J..

[23] Arici A. Potato Business; Supporting the Potato Industry Worldwide. The Search for the Ultimate CIPC Alternative Continues (Updated) 2019. https://www.potatobusiness.com/blog/blog-the-search-for-the-ultimate-cipc-alternative-continues-update/.

[24] Thoma J.L., Cantrell C.L., Zheljazkov V.D. (2022). Evaluation of Essential Oils as Sprout Suppressants for Potato (*Solanum tuberosum*) at Room Temperature Storage. Plants.

[25] Datir S.S., Yousf S., Sharma S., Kochle M., Ravikumar A., Chugh J. (2020). Cold storage reveals distinct metabolic perturbations in processing and non-processing cultivars of potato (*Solanum tuberosum* L.). Sci. Rep..

[26] Gikundi E.N., Buzera A.K., Orina I.N., Sila D.N. (2022). Storability of Irish Potato (*Solanum tuberosum* L.) Varieties Grown in Kenya, Under Different Storage Conditions. Potato Res..

[27] Bachir N., Haddarah A., Sepulcre F., Pujola M. (2022). Formation, Mitigation, and Detection of Acrylamide in Foods. Food Anal. Methods.

[28] Clasen B.M., Stoddard T.J., Luo S., Demorest Z.L., Li J., Cedrone F., Tibebu R., Davison S., Ray E.E., Daulhac A. (2016). Improving cold storage and processing traits in potato through targeted gene knockout. Plant Biotechnol. J..

[29] Elbashir H.A., Saeed I.K. (2014). Reconditioning of cold-stored Potato Varieties (*Solanum tuberosum*) Kondor and Markies. J. Agri-Food Appl. Sci..

[30] Pereira A.M., Petrucci K.P.d.O.S., Gomes M.d.P., Gonçalves D.N., Cruz R.R.P., Ribeiro F.C.S., Finger F.L. (2021). Quality of potato cv. Innovator submitted refrigeration and recondition. Food Sci. Technol..

[31] Pedreschi F., Mery D., Marique T., Sun D.W. (2016). Quality Evaluation and Control of Potato Chips. Computer Vision Technology for Food Quality Evaluation.

[32] Oguntowo O., Obadina A.O., Sobukola O.P., Adegunwa M.O. (2016). Effects of processing and storage conditions of cocoyam strips on the quality of fries. Food Sci. Nutr..

[33] European Potato Processors’ Association (EUPPA) (2011). Good Fries. EU. What Color Should Fried Fries Have? Colorguide. https://goodfries.eu/en/.

[34] Pedreschi F., Mery D., Bunger A., Yañez V. (2011). Computer vision classification of potato chips by color. J. Food Process Eng..

[35] National Potato Council of Kenya (2017). Potato Variety Catalogue.

[36] Abong G.O., Kabira J.N. (2011). Suitability of two established and three newly released Kenyan potato varieties for processing into crisps and French fries. Afr. J. Food Agric. Nutr. Dev..

[37] Buzera A., Gikundi E., Orina I., Sila D. (2022). Effect of Pretreatments and Drying Methods on Physical and Microstructural Properties of Potato Flour. Foods.

[38] Zommick D.H., Knowles L.O., Knowles N.R. (2014). Tuber respiratory profiles during low temperature sweetening (LTS) and reconditioning of LTS-resistant and susceptible potato (*Solanum tuberosum* L.) cultivars. Postharvest Biol. Technol..

[39] Kumar D., Ezekiel R. (2005). Changes in sugar content and processing quality of potatoes during storage and reconditioning. J. Food Sci. Technol..

[40] Ezekiel R., Singh B., Kumar D., Mehta A. (2007). Processing Quality of Potato Varieties Grown at Two Locations and Stored at 4, 10 and 12 °C. Potato J..

[41] Amjad A., Javed M.S., Hameed A., Hussain M., Ismail A. (2020). Changes in sugar contents and invertase activity during low temperature storage of various chipping potato cultivars. Food Sci. Technol..

[42] Abbasi S.K., Masud T., Qayyum A., Khan S.U., Abbas S., Jenks M.A. (2016). Storage Stability of Potato Variety Lady Rosetta under Comparative Temperature Regimes. Sains Malays..

[43] Wiberley-Bradford A.E., Busse J.S., Jiang J., Bethke P.C. (2014). Sugar metabolism, chip color, invertase activity, and gene expression during long-term cold storage of potato (*Solanum tuberosum*) tubers from wild-type and vacuolar invertase silencing lines of Katahdin. BMC Res. Notes.

[44] Sasaki T.D., Perez K., Himoto J.I., Itoh K. (2004). Effects of Reconditioning on the Quality of Different Processing Potato Cultivars after Low Temperature Storage. Food Preserv. Sci..

[45] Kedia P., Kausley S.B., Rai B. (2022). Development of kinetic models for prediction of reducing sugar content in potatoes using literature data on multiple potato varieties. LWT.

[46] Salehi F. (2019). Color changes kinetics during deep fat frying of kohlrabi (*Brassica oleracea var. gongylodes*) slice. Int. J. Food Prop..

[47] Pedreschi F., Mery D., Marique T., Sun D.W. (2008). Quality Evaluation and Control of Potato Chips and French Fries. Computer Vision Technology for Food Quality Evaluation.

[48] Ngobese N.Z., Workneh T.S., Alimi B.A., Tesfay S. (2017). Nutrient composition and starch characteristics of eight European potato cultivars cultivated in South Africa. J. Food Compos. Anal..

[49] Al-Dairi M., Pathare P.B., Al-Yahyai R. (2021). Effect of postharvest transport and storage on color and firmness quality of tomato. Horticulturae.

[50] Karabulut Gencer B., Acar E., Tarcın B. (2023). Evaluation of shade matching in the repair of indirect restorative materials with universal shade composites. Eur. Oral Res..

[51] Sobol Z., Jakubowski T., Nawara P. (2020). The Effect of UV-C Stimulation of Potato Tubers and Soaking of Potato Strips in Water on Color and Analyzed Color by CIE L*a*b*. Sustainability.

[52] Antonic B., Dordevic D., Buchtova H., Tremlova B., Dordevic S., Kushkevych I. (2023). French Fries’ Color and Frying Process in Relation to Used Plant Oils. Processes.

[53] Yang Y., Achaerandio I., Pujolà M. (2016). Influence of the frying process and potato cultivar on acrylamide formation in French fries. Food Control.

[54] Jaiswal S., Paul K., Raman K.V., Tyagi S., Saakre M., Tilgam J., Bhattacharjee S., Vijayan J., Mondal K.K., Sreevathsa R. (2023). Amelioration of cold-induced sweetening in potato by RNAi mediated silencing of StUGPase encoding UDP-glucose pyrophosphorylase. Front. Plant Sci..

[55] Abong G.O., Okoth M.W., Karuri E.G., Kabira J.N., Mathooko F.M. (2009). Levels of reducing sugars in eight Kenyan potato cultivars as influenced by stage of maturity and storage conditions. J. Anim. Plant Sci..

[56] Vaitkevičienė N., Jarienė E., Kulaitien J., Levickienė D. (2022). The Physico-Chemical and Sensory Characteristics of Coloured-Flesh Potato Chips: Influence of Cultivar, Slice Thickness and Frying Temperature. Appl. Sci..

[57] Abong G., Ogolla J., Michael O., Jackson K., Paul K. (2023). Effect of frying temperature and slice thickness on sensory attributes and acrylamide content in potato crisps of selected Kenyan varieties. East Afr. J. Sci. Technol. Innov..

[58] Gupta S.K., Crants J. (2019). Identification and impact of stable prognostic biochemical markers for cold-induced sweetening resistance on selection efficiency in potato (*Solanum tuberosum* L.) breeding programs. PLoS ONE.

[59] Jin Y.-I., Park K.-H., Chang D.-C., Cho J.-H., Cho K.-S., Im J.-S., Hong S.-Y., Kim S.-J., Nam J.-H., Sohn H.-B. (2016). Factors Influencing the Acrylamide Content of Fried Potato Products. Korean J. Environ. Agric..

